# Novel *RICTOR* amplification harbouring entities: FISH validation of *RICTOR* amplification in tumour tissue after next-generation sequencing

**DOI:** 10.1038/s41598-023-46927-x

**Published:** 2023-11-10

**Authors:** Dániel Sztankovics, Ildikó Krencz, Dorottya Moldvai, Titanilla Dankó, Ákos Nagy, Noémi Nagy, Gábor Bedics, András Rókusz, Gergő Papp, Anna-Mária Tőkés, Judit Pápay, Zoltán Sápi, Katalin Dezső, Csaba Bödör, Anna Sebestyén

**Affiliations:** 1 Department of Pathology and Experimental Cancer Research, Semmelweis University, Üllői út 26, 1085 Budapest, Hungary; 2 HCEMM-SE Molecular Oncohematology Research Group, Department of Pathology and Experimental Cancer Research, Semmelweis University, Üllői út 26, 1085 Budapest, Hungary; 3 Department of Pathology, Forensic and Insurance Medicine, Semmelweis University, Üllői út 93, 1091 Budapest, Hungary

**Keywords:** Cancer genetics, Cancer screening, Molecular biology, Oncology

## Abstract

Alterations in mTOR signalling molecules, including *RICTOR* amplification, have been previously described in many cancers, particularly associated with poor prognosis. In this study, *RICTOR* copy number variation (CNV) results of diagnostic next-generation sequencing (NGS) were analysed in 420 various human malignant tissues. *RICTOR* amplification was tested by Droplet Digital PCR (ddPCR) and validated using the “gold standard” fluorescence in situ hybridisation (FISH). Additionally, the consequences of Rictor protein expression were also studied by immunohistochemistry. *RICTOR* amplification was presumed in 37 cases with CNV ≥ 3 by NGS, among these, 16 cases (16/420; 3.8%) could be validated by FISH, however, ddPCR confirmed only 11 *RICTOR*-amplified cases with lower sensitivity. Based on these, neither NGS nor ddPCR could replace traditional FISH in proof of *RICTOR* amplification. However, NGS could be beneficial to highlight potential *RICTOR*-amplified cases. The obtained results of the 14 different tumour types with FISH-validated *RICTOR* amplification demonstrate the importance of *RICTOR* amplification in a broad spectrum of tumours. The newly described *RICTOR*-amplified entities could initiate further collaborative studies with larger cohorts to analyse the prevalence of *RICTOR* amplification in rare diseases. Finally, our and further work could help to improve and expand future therapeutic opportunities for mTOR-targeted therapies.

## Introduction

The mammalian target of rapamycin (mTOR) has multifunctional effects on several cellular processes of tumours (e.g. proliferation, growth, motility, protein synthesis, transcription and autophagy) by integrating signals from complex cellular networks, including metabolic adaptation mechanisms. Alterations in the mTOR signalling genes are common in malignancies (e.g. high frequency of *PIK3CA*, *PTEN* and *TSC1/2* mutations)^1^. The altered activity of two complexes, mTOR complex 1 (mTORC1) and mTOR complex 2 (mTORC2), has been described in many cancers, especially in patients with unfavourable prognoses^2,3^.

mTORC2 plays an essential role in cell differentiation, survival, growth, migration and maintenance of the actin cytoskeleton, mainly through phosphorylation of Akt (at Ser473), SGK1 and PKCα^4^. Rictor (rapamycin-insensitive companion of mTOR) is a characteristic actin co-ordinating scaffold protein of the mTORC2—instead of Raptor in the mTORC1—and its primary function is to assemble the complex. Regarding the function of mTOR complexes, it has been hypothesised that the *RICTOR* amplification could contribute to cancer progression and metastasis, through mTORC2-mediated functions in the regulation of cancer-related biochemical pathways (e.g. Wnt/β-catenin and MAPK/ERK pathways)^5^.

*RICTOR* amplification results in an increased expression of the Rictor protein and is usually associated with an increased mTORC2 complex activity. Moreover, *RICTOR* amplification or overexpression has significant clinical importance and is associated with poor prognosis and shorter overall survival in certain neoplasms^6^. Based on mutation databases, *RICTOR* amplification, a known mTOR pathway-activating oncogenic alteration, occurs in ~ 5% of certain solid tumours (e.g. lung, melanoma, endometrial, colorectal and gastric cancers). The significance of *RICTOR* amplification or Rictor protein overexpression has been described in the lung^7,8^, breast^9,10^, skin^11^ and gastrointestinal cancers^12–16^. Additionally, increased mTORC2 activity, could offer alternative therapeutic advantages and allow for personalised therapy as a general consequence of *RICTOR* amplification and their associated signalling alterations.

Over the last few decades, clinical phase trials examining the administration of phosphoinositide 3-kinase (PI3K)/mTOR pathway inhibitors have demonstrated little significant success; therefore, only a limited number of PI3K/mTOR inhibitors have been introduced into clinical practice. Even though rapamycin, an eponymous inhibitory molecule of mTOR kinase, has been isolated from microbial-derived antibiotics and was first introduced in 1975^17^. Rapamycin (or sirolimus) and rapalogs (e.g. everolimus, temsirolimus) inhibit mTORC1 activity. Many studies have shown that high mTORC2 activity and/or Rictor overexpression may correlate with therapy (including rapalog) resistance. Certain mTORC1/2 (both mTORC1 and mTORC2) and dual (both mTOR and other kinase) inhibitors have already been developed and involved in clinical phase II trials (such as vistusertib and sapanisertib). These inhibitors have shown promising results in lung cancer and other solid tumours^18,19^. Most of these inhibitors are currently used in personalised and/or combined therapies (with conventional chemotherapy/radiotherapy); the development of these inhibitors and their administration are still ongoing^20^.

Targeted therapies need reliable molecular markers to predict sensitivity. *RICTOR* gene amplification, other rare *RICTOR* mutations, epigenetic and/or signalling alterations may initiate over-representation of the mTORC2 complex linked to mTORC2 hyperactivity. These could be predictive markers for the PI3K/mTOR/Akt signalling inhibitor therapy. The above-summarised alterations can be tested using various methods: a. fluorescence in situ hybridisation (FISH) as a “gold standard” diagnostic method to identify *RICTOR* amplification^8,21,22^; b. identification of *RICTOR* sequence and copy number variations (CNVs) using sequencing (e.g. next-generation sequencing—NGS) and Droplet Digital PCR (ddPCR); c. analyses of Rictor expression or mTORC2 activity by Rictor and Phospho-Ser473-Akt (an activated mTORC2 target protein) immunohistochemistry (IHC). A combination of these established technologies can provide a complete analytical platform to determine genetic and protein expression alterations of *RICTOR* and their signalling consequences.

Our study aimed to validate potential *RICTOR* amplification detected by NGS in different malignant tumours using FISH, ddPCR; and compare the detected *RICTOR* amplification with in situ Rictor and Phospho-Ser473-Akt protein expressions obtained by IHC.

## Methods

### Case selection

Between 2018 and 2022, formalin-fixed and paraffin-embedded (FFPE) tumour tissue samples from 420 patients (mainly digestive system tumours, central nervous system tumours, soft tissue and bone tumours; see Table 1, Supplementary Table 1 for details) were sequenced using diagnostic NGS at the Department of Pathology and Experimental Cancer Research, Semmelweis University (Budapest, Hungary).

**Table 1:** Tumour type categorisation (regarding the latest WHO Classification of Tumours, 5th edition) of next-generation sequenced malignancies diagnosed between 2018 and 2022 at the Department of Pathology and Experimental Cancer Research, Semmelweis University.

Main tumour types	Case no.	Distribution (%)
Breast tumours	25	6.0
Central nervous system tumours	82	19.5
Digestive system tumours	89	21.2
Female genital tumours	52	12.4
Head and neck tumours	6	1.4
Other tumours	8	1.9
Skin tumours	4	1.0
Soft tissue and bone tumours	69	16.4
Thoracic tumours	34	8.1
Tumours of endocrine organs	35	8.3
Tumours of haematopoietic and lymphoid tissues	6	1.4
Urinary and male genital tumours	10	2.4
Total	420	100

The detailed case distribution of the main groups is shown in Supplementary Table 1.

### Next-generation sequencing

Diagnostic NGS was performed using previously described methods^23^. Before DNA isolation tumour cell percentages (TC%) of studied samples were estimated by pathologists (samples with TC% < 20% were excluded). Genomic DNA was isolated using the QIAmp DNA FFPE Tissue Kit (QIAGEN GmbH, Hilden, Germany). Illumina TruSight Oncology 500 High Throughput assay (TSO500) library preparation workflow and NGS on Illumina NextSeq 2000 platform with 101 cycles of paired-end sequencing was performed according to the manufacturer’s protocol.

Bioinformatic analysis was performed using Illumina TruSight Oncology 500 Local App v2.1. FASTQ generation was performed by BCL-convert (Binary Base Call) software after raw BCL files were downloaded. Using the Burrows-Wheeler Aligner and SAMtools utility, the sequence alignment to the hg19 reference genome was performed. Duplicate reads were removed by read collapsing analysis, marked by unique molecular identifiers. CNV calling was performed by CRAFT. Cases with normalised *RICTOR* CNV ≥ 3 were selected (the normalised CNV is 2 for cells without gene amplification and somatic cells containing the “normal”, non-amplified 2 gene copy) for our examinations.

### *RICTOR* FISH and ddPCR analyses

FISH was performed using 4-μm thick FFPE tissue sections. After deparaffinisation and citrate pre-treatment, samples were digested (37 °C, 15 min, 10% pepsin). Hybridisation was performed at 85 °C, 10 min and then at 37 °C overnight using ZytoLight SPEC *RICTOR/5q31.1* Dual Color Probe (ZytoVision GmbH; Bremerhaven, Germany). Washing steps (0.4 × SSC/0.1% NP-40 at 72 °C, 5 min and 2 × SSC/0.1% NP-40 at RT, 5 min) and DAPI (Vector Laboratories, Inc. Newark, CA, USA) counterstaining were carried out.

Representative areas were selected and evaluated by counting 30–30 nuclei in at least two regions of the tumour using the Nikon Eclipse E600 fluorescence microscope. The mean of the signal numbers per nucleus and *RICTOR/5q31.1* ratio were determined, where *RICTOR/5q31.1* ratio ≥ 2 was considered positive for *RICTOR* amplification (the *RICTOR/5q31.1* ratio is 1 in cells without gene amplification).

DNA previously isolated for NGS analysis was used for *RICTOR* gene copy number analysis by ddPCR. The PCR reaction was conducted from 50 ng extracted DNA with a *RICTOR* FAM probe (Unique Assay ID: dHsaCNS608884235, Bio-Rad Laboratories, Inc.; Hercules, CA, USA) and an *AP3B1* HEX probe (Unique Assay ID: dHsaCP2500348, Bio-Rad Laboratories, Inc.) which were described previously^21^. Droplets were generated using Bio-Rad Automated Droplet Generator, and the emulsified PCR reactions were run on Bio-Rad C1000 Touch Thermal Cycler (95 °C for 10 min, 40 cycles of 94 °C for 30 s, 60 °C for 1 min, and 98 °C for 10 min). Droplets were read and analysed using Bio-Rad QX200 Droplet Reader and QuantaSoft Software (version 1.7).

Data were normalised to the percentage of TC% of FFPE samples using the formula^24^:

$$\mathrm{Normalised}\,RICTOR/AP3B1\,\mathrm{ratio}=\frac{RICTOR/AP3B1}{\mathrm{TC\%}}\times 100$$

Normalised *RICTOR/AP3B1* ratio ≥ 2 was defined as *RICTOR* amplification (the *RICTOR/AP3B1* ratio is 1 for cells without gene amplification and somatic cells containing the “normal”, non-amplified 2 gene copy). This formula was not used below 40% tumour purity to exclude false results.

### Immunohistochemistry

IHC was performed on 4-μm thick FFPE tissue sections. After deparaffinisation, endogenous peroxidase blocking and antigen retrieval (10 mM citrate buffer, pH 6), slides were incubated with primary antibodies: anti-Rictor (Bethyl Laboratories, Inc.; Montgomery, TX, USA; A500-002A; 1:500), anti-Rictor (Cell Signaling; Danvers, MA, USA; #2140; 1:200), and anti-Phospho-Ser473-Akt (Cell Signaling, #4060; 1:100). Immunohistochemical reactions were visualised by Novolink Polymer (Leica Biosystems; Deer Park, IL, USA), followed by Dako Liquid DAB + Substrate Chromogen System (Agilent Technologies, Inc.; Santa Clara, CA, USA) and haematoxylin counterstaining.

Two independent investigators evaluated the IHC results on digitalised slides using CaseViewer software (version: 2.3, 3DHistech; Budapest, Hungary). The H-score method was used for evaluation as described previously^25^. H-scores were calculated by multiplying the percentage of positive cells by staining intensity (0, 1+, 2+, 3+). H-score < 100 was considered low, and H-score ≥ 100 was regarded as a high expression for both Rictor and Phospho-Ser473-Akt.

### Analysis of *RICTOR* alteration frequency in genomic databases

The data were collected from The Cancer Genome Atlas (TCGA, PanCancer Atlas Studies) and MSK MetTropism, downloaded via cBioPortal (www.cbioportal.org, accessed on 05 Jan 2023)^26,27^. Furthermore, CNVIntegrate, the first database that stores CNVs from cancer patients and provides statistical comparisons between copy number frequencies in different ethnic populations (www.cnvintegrate.cgm.ntu.edu.tw, accessed on 05 Jan 2023) was also used^28^.

### Statistical analysis

Statistical analyses were carried out using IBM SPSS Statistics (version 25; SPSS Inc.; Armonk, NY, USA). Spearman’s rank correlation was used to assess the correlation between the *RICTOR/5q31.1* ratio detected by FISH, the *RICTOR/AP3B1* ratio detected by ddPCR, and Rictor (both antibodies) and Phospho-Ser473-Akt expression detected by immunohistochemistry (H-scores). A *p*-value < 0.05 was considered statistically significant.

### Ethics approval and patient consent to participate statement

The archived tissue samples were used with the approval of the Hungarian Scientific Council National Ethics Committee for Scientific Research (No. 7/2006) and the Institutional Ethical Review Board (SE KREB-216/2020).

Patient consent was waived due to the retrospective nature of molecular analyses performed.

## Results

### Selecting potential *RICTOR*-amplified cases using TSO500 results

Based on TSO500 results, cases with normalised *RICTOR* CNV ≥ 3 were selected for further analyses. Potential *RICTOR* amplification was presumed in 37 (37/420) cases. The median (range) of normalised *RICTOR* CNV of these selected cases was 3.87 (3.00–7.03). Distribution of 37 cases regarding their origin: digestive system (11/37), female genital (7/37), central nervous system (6/37), soft tissue and bone (4/37), thoracic (4/37), head and neck (2/37), breast tumours (2/37) and tumours of endocrine organs (1/37). Gender and age characteristics of these patients: 54% (20/37) female and 46% (17/37) male; 65% (24/37) younger than 65 years (detailed information in Table 2).

**Table 2:** Clinicopathological characteristics of patients with tumour origin and diagnosis of 37 cases analysed in detail.

Case	Sex	Age at diagnosis (years)	Sample origin	Diagnosis	Main tumour types
#1	F	40.7	Breast	Invasive breast carcinoma of no special type (Her2- and Her2+)	Breast tumours
#2	F	47.5	Bone		
#3	F	6.1	Brain	Astrocytoma, IDH-mutant	Central nervous system tumours
#4	M	36.1	Brain	Embryonal tumour NOS	
#5	M	16.3	Brain	Germ cell tumour	
#6	M	6.3	Brain	Glioblastoma	
#7	F	52.4	Brain		
#8	M	8.0	Brain	Medulloblastoma	
#9	M	39.0	Rectum	Colorectal adenocarcinoma	Digestive system tumours
#10	F	53.1	Rectum		
#11	M	73.5	Rectum		
#12	F	66.3	Lymph node	Colorectal neuroendocrine tumour	
#13	F	69.3	Gallbladder	Gallbladder adenocarcinoma	
#14	M	26.1	Stomach	Gastric adenocarcinoma	
#15	M	56.2	Liver	Pancreatic adenocarcinoma	
#16	F	56.6	Liver		
#17	M	63.0	Pancreas		
#18	F	18.1	Pancreas	Pancreatic neuroendocrine tumour	
#19	M	80.9	Liver		
#20	F	77.8	Uterus	Endometrial carcinosarcoma	Female genital tumours
#21	F	43.7	Omentum	Squamous cell carcinoma of the cervix	
#22	F	48.3	Brain	Tubo-ovarian high-grade serous carcinoma	
#23	F	58.8	Omentum		
#24	F	66.8	Ovary		
#25	F	76.3	Omentum		
#26	F	71.7	Uterus	Undifferentiated endometrial carcinoma	
#27	M	47.7	Parotid	Acinic cell carcinoma of the salivary glands	Head and neck tumours
#28	M	30.3	Tongue	Oral squamous cell carcinoma	
#29	F	49.7	Thorax	Dedifferentiated liposarcoma	Soft tissue and bone tumours
#30	M	67.8	Retroperitoneum		
#31	M	88.3	Retroperitoneum		
#32	F	0.1	Skin	Infantile fibrosarcoma	
#33	M	54.3	Lung	Lung adenocarcinoma	Thoracic tumours
#34	F	68.0	Lung		
#35	F	70.4	Brain		
#36	M	75.7	Lymph node	Squamous cell carcinoma of the lung	
#37	M	54.6	Adrenal gland	Adrenal cortical carcinoma	Tumours of endocrine organs

*M* male, *F* female.

In 37 selected cases, FISH and ddPCR were used to test *RICTOR* amplification, while IHC was applied for evaluating Rictor and Phospho-Ser473-Akt overexpression.

FISH analysis was also applied to assess *RICTOR* amplification. The median (range) of *RICTOR/5q31.1* was 1.76 (0.94–3.29). *RICTOR* amplification (defined as *RICTOR/5q31.1* ≥ 2) were validated in 16 cases (16/37). Moreover, the median (range) of the normalised *RICTOR/AP3B1* ratio was 1.79 (0.44–5.58), and *RICTOR* amplification (defined as *RICTOR/AP3B1* ≥ 2) was observed in 11 cases (11/37) using ddPCR.

**Figure 1:**
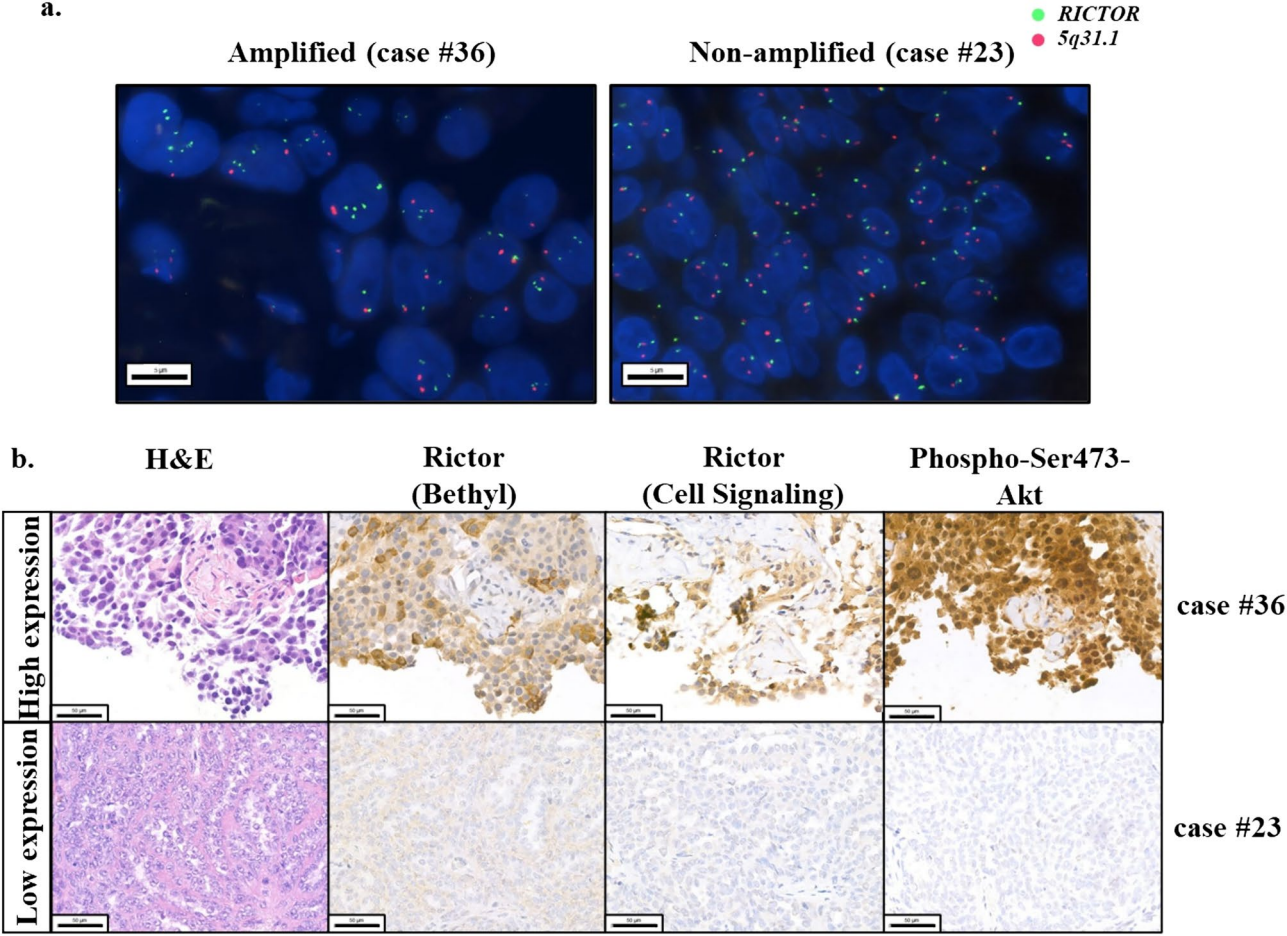
Representative images of *RICTOR*-amplified/non-amplified cases by FISH (**a**) and the high/low expressions of IHC stainings (**b**) in the studied tumour samples. The presented squamous cell carcinoma of the lung (case #36) showed *RICTOR* amplification with high Rictor and high Phospho-Ser473-Akt protein expressions. The other tubo-ovarian high-grade serous carcinoma (case #23) represents no amplification without increased Rictor and Phospho-Ser473-Akt expressions.

Expressions of Rictor and Phospho-Ser473-Akt were analysed by IHC. Two Rictor antibodies from different companies (Bethyl and Cell Signaling) were applied. High expression of Rictor was detected in 14 (14/37) and 12 (12/37) cases with Bethyl and Cell Signaling antibodies, respectively. High Phospho-Ser473-Akt expression was detected in 7 cases (7/37). Representative images of *RICTOR*-amplified/non-amplified and high/low protein expressions of IHC stainings are shown in Fig. 1.

In FISH analysis green and orange signals indicate the *RICTOR* gene and the control locus region (*5q31.1*); and the scale bars indicate 5 μm/ 50 μm for FISH and IHC, respectively.

### Analysis of NGS-predicted *RICTOR* CNVs, FISH, ddPCR results and in situ expression of characteristic mTORC2 protein (Rictor) and mTORC2 activity marker (Phospho-Ser473-Akt)

Results of *RICTOR* amplification and/or protein expression analyses were compared to *RICTOR* CNV numbers obtained by NGS. It was not surprising that: a. NGS-predicted CNV ≥ 5 has the best predictive value for *RICTOR* amplification among other CNVs (4/5); b. we found relatively more cases with positive *RICTOR* amplification by FISH among samples with NGS-predicted CNV ≥ 4 (9/12). Surprisingly, lower NGS CNV group (3.00–3.99; 25/37) cases could also be validated by FISH (7/25) or were found amplified by ddPCR (4/25). Additionally, ddPCR confirmed only 11 amplified cases among these 16 validated ones; 4 cases were associated with the lower NGS CNV group (Table 3).

**Table 3:** Comparison of FISH and ddPCR results in cases with altered *RICTOR* CNV characteristics.

NGS	FISH		ddPCR	
	Amplified	Non-amplified	Amplified	Non-amplified
25 cases with CNV 3.00–3.99 (67.5%)	7 (28%)	18 (72%)	4 (16%)	21 (84%)
7 cases with CNV 4.00–4.99 (19%)	5 (71%)	2 (29%)	4 (57%)	3 (43%)
5 cases with CNV ≥ 5.00 (13.5%)	4 (80%)	1 (20%)	3 (60%)	2 (40%)
Total = 37	16	21	11	26

IHC results were also analysed and compared with FISH-validated *RICTOR* amplification status. The combined evaluation of all three immunostainings associated well with the validated *RICTOR* amplification. 13 validated *RICTOR*-amplified cases (13/16) showed high expression with Rictor and/or Phospho-Ser473-Akt immunostainings (at least with one antibody staining) (Table 4).

**Table 4:** The combined evaluation of three immunostainings and FISH results.

Distribution of FISH results	High Rictor (Bethyl) expression	High Rictor (Cell Signaling) expression	High Rictor (Bethyl and/or Cell Signaling) expression	High Phospho-Ser473-Akt expression	High Rictor (Bethyl and/or Cell Signaling) and/or Phospho-Ser473-Akt expression
Amplified (n = 16)	9 (56%)	7 (44%)	10 (63%)	6 (38%)	13 (81%)
7 cases with CNV 3.00–3.99 (28%)	3 (43%)	4 (57%)	4 (57%)	3 (43%)	5 (71%)
5 cases with CNV 4.00–4.99 (71%)	4 (80%)	2 (40%)	4 (80%)	2 (40%)	5 (100%)
4 cases with CNV ≥ 5.00 (80%)	2 (50%)	1 (25%)	2 (50%)	1 (25%)	3 (75%)
Non-amplified (n = 21)	5 (24%)	5 (24%)	7 (33%)	1 (5%)	7 (33%)

The combined evaluation of the three different antibody stainings showed high specificity in highlighting *RICTOR* amplification. The positivity with at least one antibody staining (Rictor and/or Phospho-Ser473-Akt) compared to FISH amplification results showed 81% (13/16) specificity and 66% (14/21) sensitivity in detecting protein expression alterations associated with *RICTOR* amplification in the studied 37 cases.

Finally, we also assessed the correlations between FISH, ddPCR ratios and immunohistochemistry score results. A strong positive correlation was observed between the *RICTOR/5q31.1* ratio detected by FISH and the *RICTOR/AP3B1* ratio detected by ddPCR (*p* = 0.004, Spearman’s R = 0.491). Indeed, Rictor H-scores using antibodies from different distributors (Bethyl and Cell Signaling) also showed a strong positive correlation (*p* = 0.000, Spearman’s R = 0.639). However, Phospho-Ser473-Akt H-scores correlated only with Rictor H-scores detected by the Cell Signaling antibody (*p* = 0.002, Spearman’s R = 0.528). No significant correlation was observed between single IHC stainings and FISH or ddPCR results.

### Our NGS results and publicly available *RICTOR* alteration frequency data

We compared our NGS CNV results with publicly available datasets. We used two databases for comparative evaluation: CNVIntegrate and cBioPortal with two datasets (MSK MetTropism and TCGA PanCancer Atlas). A similar frequency of *RICTOR* gene alterations was found in several tumour types (Table 5). Our data and the MSK MetTropism dataset found the best correlation regarding tumour types (e.g. breast, hepatobiliary tract, female genital, and head and neck tumours). Among our studied cases, the highest percentage of potential *RICTOR*-amplified cases was found in central nervous system tumours (1.43%), and the lowest was in tumours of the hepatobiliary tract, stomach, endometrium and adrenal gland (0.24%).

**Table 5:** Compare the results of two publicly available databases (http://cnvintegrate.cgm.ntu.edu.tw and http://cbioportal.org) with our NGS results of *RICTOR* alteration frequencies in the studied human cancer tissues.

Tumour types		CNVintegrate (n = N/A)	cBioPortal		NGS result of our studied cases (n = 420)
			MSK MetTropism (n = 25,775)	TCGA PanCancer Atlas studies (n = 10,967)	
Breast tumours		1.40%	0.27%	1.12%	0.48%
Central nervous system tumours		0.09%	–	0.20%	1.43%
Digestive system tumours	Hepatobiliary tract	5.31%	0.22%	0.82%	0.24%
	Large intestine	0.52%	1.87%	0.68%	0.95%
	Oesophagus	7.31%	2.32%	6.61%	–
	Pancrease	0.24%	0.40%	0.54%	1.19%
	Stomach	4.99%	1.24%	-	0.24%
Female genital tumours	Cervix	7.03%	-	5.12%	0.48%
	Endometrium	1.34%	0.30%	2.42%	0.24%
	Ovary	3.58%	0.59%	5.24%	0.95%
Head and neck tumours		5.33%	0.73%	4.06%	0.48%
Skin tumours		2.04%	0.79%	2.45%	–
Soft tissue and bone tumours		4.73%	3.02%	4.74%	0.95%
Thoracic tumours	Lung	11.64%	6.80%	9.42%	0.95%
	Pleural mesothelioma	1.85%	0.41%	–	–
Tumours of endocrine organs	Adrenal gland	1.87%	–	5.62%	0.24%
	Thyroid	0.20%	–	–	–
Tumours of haematopoietic and lymphoid tissues		0.12%	–	2.08%	–
Urinary and male genital tumours	Bladder	6.21%	1.64%	5.39%	–
	Kidney	0.29%	–	0.39%	–
	Prostate	0.19%	0.32%	0.41%	–

*N/A* not available.

It is important to note that the three databases evaluated *RICTOR* alteration frequency differently. CNVIntegrate (unknown case number; data from the COSMIC dataset) considers an NGS CNV ≥ 2 as an amplified case. The TCGA PanCancer Atlas (n = 10,967) also rates putative copy number alterations ≥ 2 and the MSK MetTropsim (n = 25,775) above 2.29. In our study, cases (n = 37) with CNV ≥ 3 were considered to be set for evaluation.

### Genes with altered copy numbers that co-occur with *RICTOR* CNV

In the validated *RICTOR* amplification cases, several additional amplifications were detected by FISH for diagnostic purposes. These cases carried some further amplifications. The co-detected amplifications were as follows: 1 case with *ERBB2* amplification, 1 case with *EGFR* amplification, 1 case with *ALK* amplification, 1 case with *ERBB2* amplification and *CDK4* amplification, and 1 case with co-occurring *ERBB2*, *MET*, *ALK* and *CDK4* amplification.

The NGS results also highlighted some potentially co-occurring altered genes (with CNV ≥ 3) in the validated *RICTOR* amplification cases. These are as follows: *AKT2* (3/16), *BRCA1* (1/16), *CCND1* (5/16), *CCNE1* (2/16), *CDK4* (3/16), *CDK6* (2/16), *EGFR* (3/16), *ERBB2* (3/16), *ERBB3* (1/16), *ERCC1* (1/16), *ERCC2* (1/16), *ESR1* (1/16), *FGF3* (3/16), *FGF4* (1/16), *FGF10* (2/16), *FGF19* (2/16), *FGFR1* (3/16), *FGFR3* (2/16), *FGFR4* (3/16), *JAK2* (1/16), *KIT* (2/16), *KRAS* (2/16), *MDM2* (1/16), *MDM4* (4/16), *MET* (3/16), *MYC* (8/16), *MYCL* (1/16), *NMYC* (1/16), *PDGFRA* (2/16), *PIK3CA* (2/16), *PIK3CB* (1/16), *RET* (2/16), *RPS6KB1* (2/16), *TFRC* (1/16). Among these, *FGFR4* and *FGF10* are also located on chromosome 5, and the distance between *RICTOR* (5p13.1) and *FGF10* (5p12) genes is relatively low.

### Previously undescribed tumour types with *RICTOR* amplification

We could validate *RICTOR* amplification in 6 main tumour types with 14 diagnoses (Table 6). There were several tumour diagnoses (n = 10) where our FISH analyses were the first to validate *RICTOR* amplification. Based on mTOR activity alterations (Rictor overexpression or increased *RICTOR* CNV, without validated amplification), many of these tumour types have been identified by others as potential mTOR inhibitor targets. These are the following entities: Her2− invasive breast carcinoma of no special type, astrocytoma, glioblastoma, gallbladder adenocarcinoma, pancreatic neuroendocrine tumour, endometrial carcinosarcoma, undifferentiated endometrial carcinoma, oral squamous cell carcinoma, dedifferentiated liposarcoma, squamous cell carcinoma of the lung. Additionally, mTOR/Rictor expression and genetic alterations (e.g. amplification) have not been previously studied and described in pancreatic neuroendocrine tumours and endometrial carcinosarcomas, these are newly validated *RICTOR*-amplified entities in our study. These cases showed *RICTOR* amplification by FISH and ddPCR; case #19 with pancreatic neuroendocrine tumour (*RICTOR*/*5q31.1* ratio was 2.47 and normalised *RICTOR/AP3B1* ratio was 4.83) and case #20 with endometrial carcinosarcoma (*RICTOR/5q31.1* ratio was 2 and normalised *RICTOR/AP3B1* ratio was 3.21), as well.

**Table 6:** Our *RICTOR* amplification findings—highlighting new tumour types—and associated previously published *RICTOR* gene/expression alterations.

Main tumour types	Diagnosis	Potential *RICTOR*-amplified cases by NGS/validated *RICTOR*-amplified cases by FISH in our study	Representative publications about *RICTOR* amplification and/or Rictor overexpression (numbers indicate references)	
			Gene level	Protein level
Breast tumours	Invasive breast carcinoma of no special type (Her2− and Her2+)^**#**^	2/1 (50%)	N/A	^10,29–31^
Central nervous system tumours	Astrocytoma, IDH-mutant^**#**^	1/1 (100%)	N/A	^32^
	Embryonal tumour NOS	1/0 (0%)	N/A	N/A
	Germ cell tumour	1/0 (0%)	N/A	N/A
	Glioblastoma^**#**^	2/1 (50%)	N/A	^33,34^
	Medulloblastoma	1/0 (0%)	N/A	^35^
Digestive system tumours	Colorectal adenocarcinoma*	3/1 (33%)	^22^	^22,36–39^
	Colorectal neuroendocrine tumour	1/0 (0%)	N/A	N/A
	Gallbladder adenocarcinoma^**#**^	1/1 (100%)	^40^	N/A
	Gastric adenocarcinoma*	1/1 (100%)	^22,41^	^15,16,22,41^
	Pancreatic adenocarcinoma*	3/1 (33%)	^22^	^13,42^
	**Pancreatic neuroendocrine tumour**^**#**^	2/1 (50%)	N/A	N/A
Female genital tumours	**Endometrial carcinosarcoma**^**#**^	1/1 (100%)	N/A	N/A
	Tubo-ovarian high-grade serous carcinoma	4/0 (0%)	N/A	N/A
	Squamous cell carcinoma of the cervix	1/0 (0%)	N/A	N/A
	Undifferentiated endometrial carcinoma^**#**^	1/1 (100%)	N/A	^43^
Head and neck tumours	Acinic cell carcinoma of the salivary glands	1/0 (0%)	N/A	N/A
	Oral squamous cell carcinoma^**#**^	1/1 (100%)	N/A	^44–46^
Soft tissue and bone tumours	Dedifferentiated liposarcoma	3/1 (33%)	N/A	^47^
	Infantile fibrosarcoma	1/0 (0%)	N/A	^48^
Thoracic tumours	Lung adenocarcinoma*	3/3 (100%)	^18,22,41^	^49^
	Squamous cell carcinoma of the lung^**#**^	1/1 (100%)	^50^	N/A
Tumours of endocrine organs	Adrenal cortical carcinoma	1/0 (0%)	N/A	N/A

*N/A* not available.

*Diagnoses where *RICTOR* amplification was detected by others using FISH.

^#^Diagnoses with FISH-validated *RICTOR* amplification, where *RICTOR* amplification has not yet been described by others using FISH; bold-newly validated and previously not described (neither at gene nor protein level) diagnoses with *RICTOR* amplification.

## Discussion

The role of molecular diagnostics has recently increased significantly in personalised, molecular-targeted therapy. Sequencing of genomic regions (or even the entire genome) can reveal genomic alterations, confirm base substitutions, deletions and insertions; and highlight potential copy number changes, and gene rearrangements. Comprehensive genetic profiling using NGS requires special attention in pathology practice and laboratory work. These molecular diagnostic results are associated with many challenging difficulties. The validation and interpretation of NGS results are time-consuming and require additional costs, which are critical for clinical treatment decisions^51^. Besides the well-known targetable genetic alterations, it would be helpful to highlight certain tumour types where some additional targets can be used regarding available NGS results and their validated targets.

In our work, NGS revealed *RICTOR* CNVs were further analysed in various tumour samples, and *RICTOR* amplified cases were validated by the “gold standard” FISH, and studied by other molecular and in situ analyses. Potential *RICTOR* amplification was presumed in 37 cases by NGS and validated in 16 cases by FISH. ddPCR was less sensitive, confirming only 11 *RICTOR*-amplified cases. Based on these results, the specificity and sensitivity of NGS and ddPCR could not allow for replacing the FISH but could help highlight potential *RICTOR*-amplified cases. The 14 different cancer diagnoses described with validated *RICTOR* amplification underline its importance in a wide range of tumours, including some rare malignancies. The already published research works were reviewed to collect data (and referred to in Table 6) where *RICTOR* amplification or overexpression was detected and described in different tumour types. Regarding these, our newly validated *RICTOR* amplification results highlighted the following: a. there are several associations between our results and the available data about *RICTOR* alterations; b. we could validate *RICTOR* amplification first by FISH in several main tumour types, where previously potentially targetable Rictor overexpression or increased NGS CNV had been highlighted; c. among the 16 FISH-validated cases, pancreatic neuroendocrine tumour and endometrial carcinosarcoma were described for the first time in our study as new, previously not studied *RICTOR*-amplified entities (neither the *RICTOR* gene alteration nor the Rictor expression changes have been previously studied in these tumour types).; d. interestingly, *RICTOR* amplification was not validated in 4 presumed tubo-ovarian high-grade serous carcinoma cases, e. *RICTOR* amplification was validated in all enrolled lung adenocarcinomas (n = 3).

Our screened patient population had an unusual distribution regarding the Hungarian centralisation, an above-average prevalence of rare malignancies, central nervous system tumours (CNS), as well as soft tissue and bone tumours and paediatric cases (all paediatric tumour samples are sent to Semmelweis University). It should be noted that only a few lung cancers (e.g. lung adenocarcinoma, squamous cell carcinoma of the lung) were included in our recent study (most lung adenocarcinoma cases are tested on Illumina TruSight Tumor 170 panel). We have previously contributed to *RICTOR* amplification analysis of small cell lung carcinoma cases, and the Hungarian lung adenocarcinoma cohort has already been characterised for mTORC1/2 activity by our research^8,49^. Among the paediatric *RICTOR* CNV-selected patients (5/37), 2 CNS tumour cases were validated with *RICTOR* amplification. This could be particularly interesting in childhood glioblastoma and astrocytoma regarding described mTOR and mTORC2 hyperactivity in CNS malignancies^52,53^. Phase trials showed low effectiveness of PI3K/Akt/mTOR inhibitors^54^. However, neither mTORC2 complex hyperactivity nor *RICTOR* amplification has been studied before treatment decisions.

Regarding the newly validated *RICTOR*-amplified cases, the potential role of high mTORC2 activity has already been described in glioblastoma, astrocytoma and invasive breast carcinoma of no special type. Targeting PI3K/AKT/mTOR signalling pathway mutations with inhibitors could benefit patients diagnosed with these entities^10,55–57^. Additionally, mTOR inhibitors and specific targeting of Rictor modifications have been very effective in experimental glioblastoma models^58–60^. In ~ 50–60% of gallbladder adenocarcinomas, mTOR hyperactivity has also been described in association with poor prognosis (but *RICTOR* alterations were not analysed)^61–63^. The revealed undifferentiated endometrial carcinoma case in our cohort underlines the importance of previously described mTORC2 activity as a risk/prognostic factor^43^ and a phase I study with sapanisertib showing anti-tumour activity in certain renal and endometrial cancer cases^64^. *RICTOR*-amplified cases of dedifferentiated liposarcoma, oral squamous cell carcinoma and lung squamous cell carcinoma confirm the importance of rapalogs and dual inhibitors as treatment options in these malignancies^65–67^. Additionally, *PIK3CA* and/or *EGFR* amplification and Rictor overexpression in head and neck squamous cell carcinoma and lung squamous cell carcinoma, and the *RICTOR* amplification detected in our study are associated with the fact that mTOR inhibitors are under investigation in these tumours^45,68^.

There has been some success with mTOR inhibitors in treating pancreatic neuroendocrine tumours and heavily pre-treated endometrial carcinosarcomas in clinical trials. Still, no data is specific to *RICTOR* amplification in these malignancies^19,69–72^. Additionally, mTOR inhibitors have been tested in combination with gemcitabine^73^, and in association with *PIK3CA* mutations, samotolisib showed some efficacy and a manageable safety profile in endometrial carcinosarcomas^74^. *RICTOR* gene and expression alterations have not been previously reported in pancreatic neuroendocrine tumours and endometrial carcinosarcomas. Therefore, our is the first study about high mTORC2 activity with validated *RICTOR* amplification in these entities.

Combined with the validated *RICTOR* amplification, increased expression of Rictor and Phospho-Ser473-Akt were confirmed as targetable signalling alterations. There were only 3 cases (3/16) where FISH-validated *RICTOR* amplification was not associated with altered Rictor and/or Phospho-Ser473-Akt protein levels. Possible causes of these discrepancies could be explained by epigenetic modifications and complex biological mechanisms (e.g. post-translational regulatory mechanisms)^4,75–83^; and further technical issues (e.g. biopsy sample handling, antibody specificity and sensitivity differences). These results suggest that the applied immunostainings could be helpful in pre-screening cases before *RICTOR* FISH analysis. Both *RICTOR* FISH and the above-mentioned IHCs would be beneficial in better patient selection before targeting mTOR/mTORC1 hyperactivity. Corresponding to our findings, some studies have suggested that *RICTOR* amplification and/or Rictor overexpression might be predictive markers to identify cancer patients who will respond to dual mTORC1/2 inhibitors^18,84^. Besides the allosteric mTOR inhibitors (sirolimus, everolimus and temsirolimus), numerous PI3K/Akt/mTOR inhibitors have been under development. mTOR kinase inhibitors (e.g. sapanisertib, vistusertib); dual PI3K, mTOR inhibitors (e.g. paxalisib, samotolisib) and Akt inhibitors (e.g. ipatasertib, capivasertib) are all in active phase 2 and 3 trials^85^. Nevertheless, the recent monotherapies have low overall survival benefits. The selection of optimal mTOR inhibitor combinations with chemo/radiotherapy in selected patient populations needs to be considered, however, increased toxicity of combination therapy may limit the success of these treatments^86^.

A biomarker-driven umbrella study which selected patients based on the presence of *RICTOR* amplification demonstrates a promising strategy for personalised treatments and identifies patients who are most likely to respond to targeted therapy (vistusertib)^87^. These underline the significance of validated *RICTOR* amplification targets in small cell lung cancers or other malignancies.

In conclusion, there is no consensus on the actual predictive value of the NGS-predicted CNV. Our results could highlight *RICTOR* amplification in a wide range of heavily pre-treated and/or difficult-to-treat malignancies. Our findings also demonstrate the importance of *RICTOR* amplification validation, especially in cases where tumours may have been inappropriately studied using conventional techniques. Additionally, our *RICTOR* amplification validated cases with 3 ≤ CNV < 4 characteristics could highlight that lower CNV scores should be also considered in further analyses. Finally, *RICTOR* FISH and IHC stainings (Rictor, Phospho-Ser473-Akt) are highly reliable and cost-effective validation methods before the administration of targeted therapy using mTORC1/2 inhibitors in various cancers.

### Supplementary Information

Supplementary Table 1.

## Data Availability

Publicly available datasets were analysed in this study; The Cancer Genome Atlas (TCGA, PanCancer Atlas Studies) and MSK MetTropism, downloaded via cBioPortal (www.cbioportal.org, accessed on 05 Jan 2023). Furthermore, we also used CNVIntegrate (www.cnvintegrate.cgm.ntu.edu.tw, accessed on 05 Jan 2023). Additionally, the original contributions presented in the study are available in the article/Supplementary Material provided. For further inquiries, please contact the corresponding author.
