# Supplementary material for: Novel RICTOR amplification harbouring entities: FISH validation of RICTOR amplification in tumour tissue after next-generation sequencing
Source: Sci Rep. 2023 Nov 10;13:19610. doi: 10.1038/s41598-023-46927-x (PMC10638425; doi:10.1038/s41598-023-46927-x)
Supplement: Supplementary file 1 — Supplementary Table 1. [file 41598_2023_46927_MOESM1_ESM.pdf]

Novel *RICTOR* Amplification Harbouring Entities - FISH Validation of *RICTOR* Amplification in Tumour Tissue After Next-Generation Sequencing

Authors

Dániel Sztankovics, Ildikó Krencz, Dorottya Moldvai, Titanilla Dankó, Ákos Nagy, Noémi Nagy, Gábor Bedics, András Rókusz, Gergő Papp, Anna-Mária Tőkés, Judit Pápay, Zoltán Sápi, Katalin Dezső, Csaba Bödör, Anna Sebestyén

Supplementary Table 1. The detailed case distribution of the main tumour types (regarding the latest WHO Classification of Tumours, 5th edition) of next-generation sequenced malignancies diagnosed between 2018 and 2022 at the Department of Pathology and Experimental Cancer Research, Semmelweis University.

Main Tumour Types	Diagnosis	Case no.
**Breast Tumours**	Carcinoma with apocrine differentiation	1
	Invasive breast carcinoma of no special type	24
	**Total**	**25**
**Central Nervous System Tumours**	Astrocytoma, IDH-mutant	9
	Craniopharyngioma	3
	Desmoplastic infantile astrocytoma	1
	Diffuse low-grade glioma, NOS	1
	Dysembryoplastic neuroepithelial tumour	1
	Embryonal tumour, NOS	3
	Ependymoma	5
	Ganglioglioma	1
	Germ cell tumour	3
	Glioblastoma	34
	High-grade glioma, NOS	1
	Langerhans cell histiocytosis	1
	Medulloblastoma	11
	Meningioma	1
	Pilocytic astrocytoma	6
	Primary intracranial sarcoma, NOS	1
	**Total**	**82**

**Digestive System Tumours**	Cholangiocarcinoma	10
	Colorectal adenocarcinoma	45
	Colorectal neuroendocrine tumour	1
	Gallbladder adenocarcinoma	3
	Gastric adenocarcinoma	3
	Germ cell tumour	2
	Hepatoblastoma	1
	Hepatocellular carcinoma	5
	Oesophageal squamous cell carcinoma	1
	Pancreatic adenocarcinoma	13
	Pancreatic neuroendocrine tumour	4
	Ampullary carcinoma	1
	**Total**	**89**
**Female Genital Tumours**	Choriocarcinoma	1
	Clear cell carcinoma of the cervix	1
	Clear cell carcinoma of the ovary	1
	Clear cell carcinoma of the vulva	1
	Endometrial carcinosarcoma	2
	Endometrioid endometrial carcinoma	9
	High-grade endometrial stromal sarcoma	1
	Immature teratoma	3
	Endometrial large cell neuroendocrine carcinoma	1
	Neuroendocrine tumour of gynaecological origin	1
	Serous borderline tumour	1
	Serous endometrial carcinoma	1
	Small cell carcinoma of the ovary, hypercalcaemic type	2
	Squamous cell carcinoma of the cervix	4
	Tubo-ovarian high-grade serous carcinoma	20
	Undifferentiated endometrial carcinoma	1
	Uterine leiomyosarcoma	2
	**Total**	**52**
**Head and Neck Tumours**	Acinic cell carcinoma of the salivary glands	1
	Adenoid cystic carcinoma of the salivary glands	3
	Low-grade malignant soft tissue tumour, NOS	1
	Oral squamous cell carcinoma	1
	**Total**	**6**
**Other Tumours**	Carcinoma of unknown primary	4
	Mucinous adenocarcinoma of unknown origin	3
	Retinoblastoma	1
	**Total**	**8**

**Skin Tumours**	Melanoma	4
	**Total**	**4**
**Soft Tissue and Bone Tumours**	Alveolar rhabdomyosarcoma	4
	Alveolar soft part sarcoma	1
	Angiomatoid fibrous histiocytoma	1
	Angiosarcoma	3
	Dedifferentiated liposarcoma	4
	Dermatofibrosarcoma protuberans	1
	Desmoid fibromatosis	1
	Embryonal rhabdomyosarcoma	1
	Epithelioid haemangioendothelioma	1
	Epithelioid sarcoma	3
	Ewing sarcoma	11
	Infantile fibrosarcoma	2
	Inflammatory myofibroblastic tumour	4
	Interdigitating dendritic cell sarcoma	3
	Intimal sarcoma	1
	Kaposiform haemangioendothelioma	1
	Leiomyosarcoma	1
	Myofibroblastoma	2
	Myopericytoma	1
	Nodular fasciitis	3
	Osteosarcoma	4
	PEComa	1
	Primitive myxoid mesenchymal tumour of infancy	1
	Rhabdoid tumour	3
	Sarcoma with *BCOR* genetic alterations	1
	Solitary fibrous tumour	2
	Spindle cell rhabdomyosarcoma	1
	Synovial sarcoma	2
	Undifferentiated pleomorphic sarcoma	3
	Undifferentiated small round cell sarcoma	2
	**Total**	**69**
**Thoracic Tumours**	Lung adenocarcinoma	22
	Mesothelioma	3
	Sarcomatoid carcinoma of the lung	1
	Small cell lung carcinoma	4
	Squamous cell carcinoma of the lung	4
	**Total**	**34**
**Tumours of Endocrine Organs**	Adrenal cortical carcinoma	3
	Medullary thyroid carcinoma	3

	Mixed neuroendocrine–non-neuroendocrine carcinoma	1
	Neuroblastoma	14
	Papillary thyroid carcinoma	12
	Parathyroid carcinoma	2
	**Total**	**35**
**Tumours of Haematopoietic and Lymphoid Tissues**	B-cell prolymphocytic leukaemia	1
	Chronic lymphocytic leukaemia	1
	Diffuse large B-cell lymphoma	3
	Primary mediastinal large B-cell lymphoma	1
	**Total**	**6**
**Urinary and Male Genital Tumours**	Prostatic adenocarcinoma	3
	Urachal carcinoma	4
	Urothelial carcinoma	3
	**Total**	**10**
	∑	**420**
